# Sex, size and isotopes: cryptic trophic ecology of an apex predator, the white shark *Carcharodon carcharias*

**DOI:** 10.1007/s00227-018-3343-x

**Published:** 2018-05-17

**Authors:** G. C. A. French, S. Rizzuto, M. Stürup, R. Inger, S. Barker, J. H. van Wyk, A. V. Towner, W. O. H. Hughes

**Affiliations:** 1 School of Life Sciences, University of Sussex, Brighton, BN1 9QG UK; 2 Department of Botany and Zoology, Stellenbosch University, Merriam Avenue, Stellenbosch, 7600 South Africa; 3 Environment and Sustainability Unit, University of Exeter, Penryn Campus, Penryn, Cornwall TR10 9FE UK; 4 Dyer Island Conservation Trust, Kleinbaai, South Africa

## Abstract

Demographic differences in resource use are key components of population and species ecology across the animal kingdom. White sharks (*Carcharodon carcharias*) are migratory, apex predators, which have undergone significant population declines across their range. Understanding their ecology is key to ensuring that management strategies are effective. Here, we carry out the first stable isotope analyses of free-swimming white sharks in South Africa. Biopsies were collected in Gansbaai (34.5805°S, 19.3518°E) between February and July 2015. We used Stable Isotope Bayesian Ellipsis in R and traditional statistical analyses to quantify and compare isotopic niches of male and female sharks of two size classes, and analyse relationships between isotopic values and shark length. Our results reveal cryptic trophic differences between the sexes and life stages. Males, but not females, were inferred to feed in more offshore or westerly habitats as they grow larger, and only males exhibited evidence of an ontogenetic niche shift. Lack of relationship between δ^13^C, δ^15^N and female shark length may be caused by females exhibiting multiple migration and foraging strategies, and a greater propensity to travel further north. Sharks < 3 m had much wider, and more diverse niches than sharks > 3 m, drivers of which may include individual dietary specialisation and temporal factors. The differences in migratory and foraging behaviour between sexes, life stages, and individuals will affect their exposure to anthropogenic threats, and should be considered in management strategies.

## Introduction

Patterns of resource use are a key component in the ecology of species, and such data are vital for ensuring that wildlife management and conservation measures are successful. Individual variation in resource use has been highlighted as a critical topic in further understanding species, and community ecology (Bolnick et al. 2003, 2011; Réale et al. 2010; Sih et al. 2012; Dall et al. 2012), particularly in the case of predators (Schreiber et al. 2011), and is emerging as an important facet in the study of elasmobranchs (Matich et al. 2011; Jacoby et al. 2014; Huveneers et al. 2015; Matich and Heithaus 2015; Towner et al. 2016). Ecological differences between males and females in elasmobranchs are already recognised as prevalent (Sims 2005), and form another important consideration in the understanding of their ecology, and consequently their effective management. The niche concept (Hutchinson 1957), has been recognised as a tool for quantifying resource specialisation and overlap between individuals, and species (Van Valen 1965; Kohn 1968; Cody 1974). This concept has recently been reinvigorated by construction of the isotopic niche, in which stable isotope ratios of carbon and nitrogen (in δ denomination) of study organism tissue are plotted in bivariate space (Bearhop et al. 2004; Layman et al. 2007; Newsome et al. 2007). The isotopic constituents of an animal’s tissues reflect the isotopic composition of the organisms on which they feed, with nitrogen isotopes (δ^15^N) being considered to provide reliable reflections of trophic position (Post 2002) and carbon isotopes (δ^13^C) indicating habitat use (DeNiro and Epstein 1978).

The white shark (*Carcharodon carcharias*) is a large predatory fish (Compagno 2001), and is currently listed as Vulnerable on the IUCN Red List (Fergusson et al. 2009), due to significant population declines, largely attributed to targeted overfishing and bycatch, which has resulted in relatively small contemporary populations across its range (Baum 2003; Gubili et al. 2011; Chapple et al. 2011; Blower et al. 2012; Nasby-Lucas and Domeier 2012; Towner et al. 2013). Upon reaching approximately 3 m in length, white sharks are thought to undergo an ontogenetic shift in diet, from being largely piscivorous to a greater emphasis on marine mammals (Tricas and McCosker 1984; Casey and Pratt 1985; Cliff et al. 1989; Compagno 2001; Hussey et al. 2012b). There is suggestion of individual dietary specialisation in white sharks (Estrada et al. 2006; Hussey et al. 2012b; Carlisle et al. 2012; Kim et al. 2012; Hamady et al. 2014; Pethybridge et al. 2014; Christiansen et al. 2015), evidence of individual variation in predatory behaviours (Huveneers et al. 2015; Towner et al. 2016), and sexual differences in movement patterns (Pardini et al. 2001; Anderson and Pyle 2003; Domeier and Nasby-Lucas 2007; Weng et al. 2007; Jorgensen et al. 2010; Bruce and Bradford 2012; Domeier and Nasby-Lucas 2012; Robbins and Booth 2012; Kock et al. 2013).

The South African population of white sharks has five main coastal aggregation sites (from west to east: False Bay, Gansbaai, Struisbaai, Mossel Bay and Algoa Bay). These aggregations are not genetically distinct (Andreotti et al. 2016), with sharks migrating between them, and further along the South African coast to KwaZulu-Natal (KZN), Mozambique and the Western Indian Ocean (Cliff et al. 1996; Ferreira and Ferreira 1996; Bonfil et al. 2005; Jewell et al. 2011). Some segregation by shark size is apparent between the sites, with average size typically increasing from west to east (Cliff et al. 1989, 1996; Ferreira and Ferreira 1996; Dicken 2008; Kock et al. 2013; Towner et al. 2013; Hewitt 2014; Ryklief et al. 2014). With the exception of Struisbaai, these locations are characterised by the presence of pinniped colonies (Dudley 2012). Mature females are notable by their rarity from all of these aggregations, and they have instead been documented in the more tropical waters of the Western Indian Ocean (Cliff et al. 2000; Bonfil et al. 2005).

Previous studies of diet in South African white sharks, both through gut content analysis and isotopic analyses, have been based on samples from relatively small sharks caught in the nets of a bather safety programme managed by the KZN Sharks Board (Cliff et al. 1989; Hussey et al. 2012b; Christiansen et al. 2015), and have not included an analysis of niche space. Christiansen et al. (2015) have urged that isotopic results be interpreted within a multidisciplinary framework, to obtain the most accurate and useful data from which management decisions can be deduced. Biopsy sampling provides a non-lethal method of collecting shark tissue for stable isotope analysis, which may be of particular benefit for elasmobranchs, many of which are undergoing severe population declines at a global scale and require informed conservation management (Myers and Worm 2003; Worm et al. 2013; Dulvy et al. 2014). Here, in addition to traditional statistics, we use metrics derived from stable isotope bivariate plots (Layman et al. 2007; Jackson et al. 2011) to visualise and quantify the variation in niche among potential pre- and post-ontogenetic shift male and female sharks and interpret our results in the context of published diet, telemetry, sighting and capture data, in the first isotopic study of free-swimming white sharks in South Africa.

## Methods

Tissue biopsy samples were collected from white sharks between February and July 2015 within the designated white shark cage-diving area in Gansbaai, South Africa. Collection took place from either a 9 m research catamaran or a 14 m custom-built shark cage-diving catamaran, owned and operated by the Dyer Island Conservation Trust and Marine Dynamics Shark Tours. Sharks were brought close to the vessels using fish oil chum and a salmon head bait lure. Photographs were taken for individual identification based on distinguishing marks and DARWIN dorsal fin ID software (http://darwin.eckerd.edu/). Finn Larsen Ceta darts (4 × 0.9 cm) affixed to a biopsy pole were used to excise cores of tissue, comprising muscle and dermis, from the dorsal surface of free-swimming sharks. Samples were stored immediately in ethanol.

Shark sex was classified by the presence or absence of claspers, and only samples from the 26 sharks of known sex were included in the study. Shark total length was estimated by comparison of free-swimming sharks with a 4.7 m object of known length (Kock et al. 2013; Towner et al. 2013). For the Stable Isotope Bayesian Ellipsis in R (SIBER) analyses (see below), sharks were classified as either < 3 m (six females, five males), or > 3 m (ten females, five males) to reflect pre-and post-ontogenetic shift life stages (Tricas and McCosker 1984; Casey and Pratt 1985; Cliff et al. 1989; Compagno 2001; Hussey et al. 2012b).

Twenty-six samples were prepared for stable isotope analysis. Muscle and dermis have different isotopic turnover rates, and muscle isotopic turnover can take up to 2 years (Martinez del Rio et al. 2009; Logan and Lutcavage 2010; Hussey et al. 2012a). Only muscle was used for analysis. Ethanol was removed from the tissues by blowing with nitrogen for 20 min at 30 °C using a Techne dri-block DB.2A, and samples were freeze-dried overnight. Storage of fish muscle in ethanol causes small but directionally uniform changes to δ^13^C and δ^15^N values (Arrington and Winemiller 2002), and so would not affect between-sample comparisons. Dried samples were homogenised using scissors, weighed and placed into tin capsules. Lipid and urea extraction are recommended prior to isotope analysis of elasmobranch tissues as presence of lipids, trimethylamine and urea can affect isotopic values and ratios, which precludes accurate estimation of trophic position and diet reconstruction (Fisk et al. 2002; Hussey et al. 2012c). Lipid and urea extraction were not performed, because our main aim was to perform comparative analyses within our own samples, and no effect of increasing animal size has been detected (Hussey et al. 2012c).

Stable isotope ratios were measured using continuous flow isotope ratio mass spectrometry using a Sercon Integra integrated elemental analyser and mass spectrometer. Stable isotope ratios are reported as *δ* values and expressed in ‰, according to the following: *δ X *= [(*R*_sample_/*R*_standard_) − 1] × 1000, where *X* is ^13^C or ^15^N and *R* is the corresponding ratio ^13^C/^12^C or ^15^N/^14^N, and *R*_standard_ is the ratio of the international references PDB for carbon and AIR for nitrogen. Replicate analyses of internal lab standard alanine yielded standard deviations of 0.15‰ for δ^15^N and 0.09‰ for δ^13^C. δ^13^C and δ^15^N data were averaged between the two analytical runs and tested for outliers using the package ‘Outliers’ in R statistical software version 3.3.1., which was used for all analyses (Komsta 2011; R Core Team 2016). Data points that fell outside of 95% of the normal distribution were removed to create an ‘outlier-removed’ dataset, which we believe provides useful results, despite low sample size.

General linear models (GLMs) were used to assess the relationship between outlier-removed δ^13^C and δ^15^N values, and for relationships between and shark total length (m) and sex, respectively. Models specified a Gaussian distribution and identity link function, and all two-way interactions were included in the full models. Backward stepwise elimination of variables, using Akaike Information Criterion (AIC) (Akaike 1973), and variable significance, was used to pare models. *F* values were produced by comparing full and null models in an ANOVA. Differences in median δ^13^C and δ^15^N between the sexes were analysed for both averaged and outlier-removed datasets using independent samples Mann–Whitney *U* tests, and differences in the variance of these data were tested using a Fligner–Killeen test. For the statistical analyses described above, *P* values were considered significant if ≤ 0.05. To investigate dietary specialisation, we used the pamk function in R package ‘fpc’ to determine the optimal number of clusters for a *k*-means cluster analysis of averaged δ^13^C and δ^15^N, and averaged δ^13^C and δ^15^N with outliers removed. This method uses optimum average silhouette width to suggest the number of data clusters based on medoids (Hennig 2015).

We used the SIBER package in R to compute the size and overlap of isotopic niches for < 3, and > 3 m male and female sharks and compared results produced from analyses run with averaged, and outlier-removed datasets (Jackson et al. 2011). While resultant sample sizes were low, in some cases comprising the minimum number of data points required for SIBER analysis (Jackson et al. 2011) we believe that the data still provide useful information. Isotopic niches based on δ^13^C and δ^15^N were plotted in SIBER, and values of niche size produced from estimates of small sample size corrected standard ellipse areas (SEAc) and total area (TA) of convex hulls. Bayesian estimates of standard ellipse area were generated using 10,000 repetitions and the probabilities of each demographic group (“Group A”) being smaller than the other demographic groups in turn (“Group B”) were calculated and plotted with 50, 75 and 95% credible intervals. Layman metrics were computed for each group, providing values of nitrogen range (NR), carbon range (CR), mean distance to centroid (CD), mean nearest neighbour distance (MNND), and the standard deviation of MNND (SDMNND) (Layman et al. 2007). Wider nitrogen and carbon isotope ranges suggest wider trophic diversity and a greater number of basal food sources exploited, respectively, while CD provides a metric of the average degree of trophic diversity. MNND gives a measure of trophic similarity within each group, where smaller numbers would indicate that individuals within a group have more similar ecologies, and SDMNND provides a similar measure, but less influenced by sample size. Isotopic niche overlap was calculated as the % of a group’s SEAc that overlapped with the SEAc of another group.

## Results

**Fig. 1:**
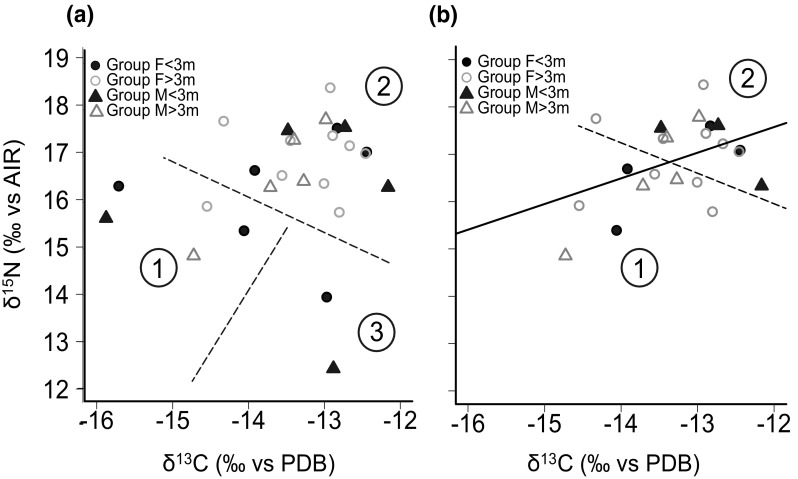
**a** *K*-means cluster analysis of averaged δ^13^C and δ^15^N data for white sharks (*Carcharodon carcharias*) from the Gansbaai aggregation separated by sex and size category: female sharks < 3 m (closed black circles *n* = 6), female sharks > 3 m (open grey circles *n* = 10), male sharks < 3 m (closed black triangles *n* = 5), and male sharks > 3 m (open grey triangles *n* = 5). Three clusters were indicated in the analysis (1, 2, 3 demarcated by a dashed line). **b** Linear regression (*y* = 0.35*x* × 19.17, *R*^2^ = 0.15, *P* = 0.043) and k-means cluster analysis results of averaged and outlier-removed δ^13^C and δ^15^N data; female sharks < 3 m (closed black circles *n* = 4), female sharks > 3 m (open grey circles *n* = 10), male sharks < 3 m (closed black triangles *n* = 3), and male sharks > 3 m (open grey squares *n* = 5); two clusters were indicated by the analysis (1 and 2, demarcated by a dashed line)

**Fig. 2:**
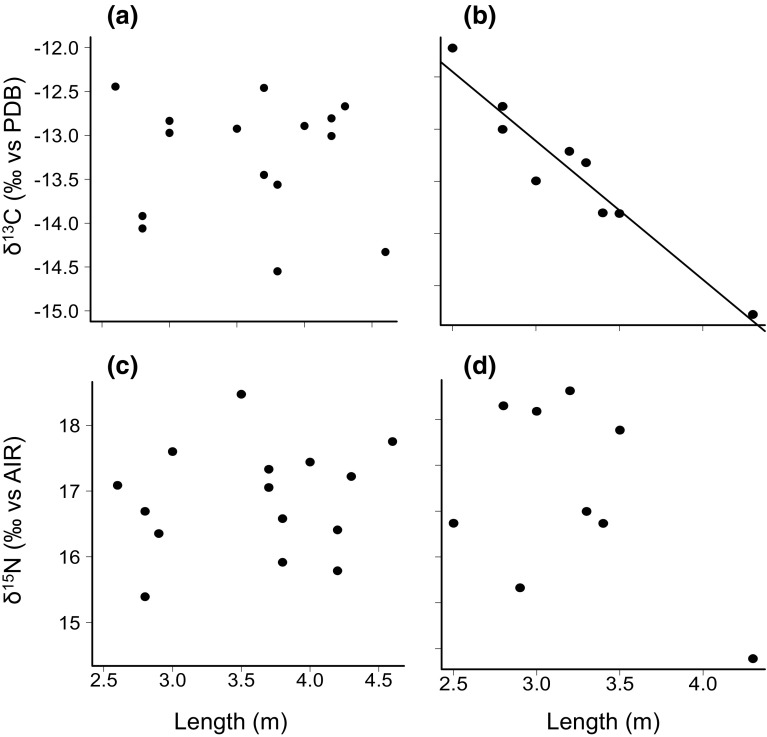
Relationships between **a** female length and δ^13^C, **b** male length and δ^13^C, **c** female length and δ^15^N, and **d** male length and δ^15^N, for white sharks sampled at the Gansbaai aggregation

Two δ^13^C and two δ^15^N outliers (each from a separate individual, all juveniles) were identified, resulting in 24 samples being included in GLM analyses, and 22 included in SIBER analyses. δ^13^C and δ^15^N values were significantly related [linear regression: *r*^2^ = 0.15, *F*(1,20) = 4.66, *P* = 0.043, confidence interval on the slope 0.01 and 0.69; Fig. 1a], with larger males in particular exhibiting a conspicuous linear trend. There was no effect on δ^15^N of shark sex or length [GLM: *F*(1,2) = 0.89, *P* = 0.24], but there was a significant interaction between the effects of shark sex and length on δ^13^C [GLM: *F*(1,2) = 3.57, *P* = 0.018]. There was no relationship between δ^13^C and female length (Fig. 2a), but it was negatively correlated with the length of male sharks (Fig. 2b). There was also no relationship between δ^15^N and female length (Fig. 2c), but while there was no significant relationship between δ^15^N and male length, there was a decreasing trend (Fig. 2d). Overall, neither δ^13^C nor δ^15^N differed between males and females (averaged data: Mann–Whitney *U* = 70, *N*1 = 10, *N*2 = 16, *P* = 0.91, and *U* = 71, *N*1 = 10, *N*2 = 16, *P* = 0.86, respectively; outlier-removed data: Mann–Whitney *U *= 86, *N*1 = 9, *N*2 = 15, *P* = 0.78, and *U* = 87, *N*1 = 9, *N*2 = 15, *P* = 0.74, respectively), and the variances of the data also did not differ between males and females for either δ^13^C or δ^15^N (averaged data, *df* = 1 in all cases: Chi-square test: *χ*^2^ < 0.001, *P* = 0.95; *χ*^2^ = 0.52, *P* = 0.47, respectively; outlier-removed data: Chi-square test: *χ*^2^ = 0.005, *P* = 0.95; *χ*^2^ = 0.516, *P* = 0.47, respectively). The pamk function revealed that paired δ^13^C and δ^15^N data split optimally into three clusters for the averaged data, heavily influenced by the inclusion of outliers (Fig. 1a). Cluster 1 comprised sharks with moderate δ^15^N, and low δ^13^C values, while cluster 2 was typified by sharks with relatively high δ^15^N and moderate to high δ^13^C, and lastly cluster 3 contained juveniles with low δ^15^N but relatively high δ^13^C values (Fig. 1a). In the outlier-removed dataset, the data split into two clusters, where sharks grouped into cluster 2 exhibited slightly higher δ^13^C and δ^15^N values than cluster 1 (Fig. 1b). The average lengths of female sharks within these clusters were almost identical (3.6 and 3.68 m, respectively) but there was a distinctive difference in the average male shark lengths of the two clusters (3.67 and 3.0 m, respectively). As this dataset was less biased by outlying data points, it likely reflects a more accurate clustering of the isotopic data within the Gansbaai aggregation.

**Fig. 3:**
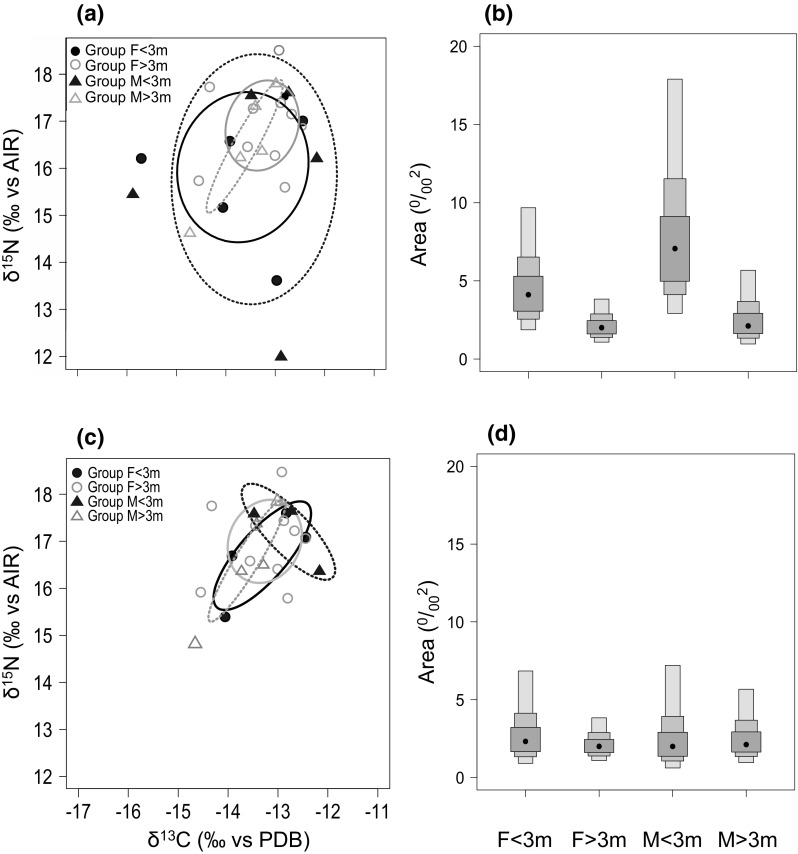
Isotopic niches of 22 white sharks sampled at the Gansbaai aggregation **a** SIBER generated biplots of averaged δ^13^C and δ^15^N values with small sample size corrected standard ellipse areas (SEAc) for female sharks < 3 m (closed black circles, solid black line *n* = 6), female sharks > 3 m (open grey circles, solid grey line *n* = 10), male sharks < 3 m (closed black triangles, dashed black line *n* = 5), and male sharks > 3 m (open grey triangles, dashed grey line *n* = 5). **b** Credible intervals (95, 75, 50%) of Bayesian estimates of SEAc for averaged δ^13^C and δ^15^N values for female sharks < 3 m, female sharks > 3 m, male sharks < 3 m, male sharks > 3 m. **c** Averaged and outlier-removed δ^13^C and δ^15^N values with small sample size corrected standard ellipse areas (SEAc), for female sharks < 3 m (closed black circles, solid black line *n* = 4), female sharks > 3 m (open grey circles, solid grey line *n* = 10), male sharks < 3 m (closed black triangles, dashed black line *n* = 3), and male sharks > 3 m (open grey triangles, dashed grey line *n* = 5). **d** Credible intervals (95%, 75%, 50%) of Bayesian estimates of SEAc for averaged and outlier-removed δ^13^C and δ^15^N values for female sharks < 3 m, female sharks > 3 m, male sharks < 3 m, male sharks > 3 m

In the averaged data, both female and male sharks > 3 m had markedly smaller isotopic niche regions than sharks < 3 m, as indicated by estimates of SEAc, TA, and probabilities generated by SIBER analysis (Tables 1, 2, Fig. 3a). Large (> 3 m) males had the smallest isotopic niche, while small (< 3 m) males had the largest, and the greatest trophic diversity (Tables 3, 4). The greatest difference in isotopic niche size was for smaller males, with the niche of male sharks < 3 m being significantly larger than that of males or females > 3 m at the 75% credible interval limit (Fig. 3b), and overlapping all other groups by 100% (Table 3). The smallest overlap in SEAc was between larger and smaller males, with males > 3 m only overlapping with 9.02% of the niche for males < 3 m. Smaller females had 1.6 times greater overlap with larger females than they did with larger males, and overlap between larger and smaller females was three times greater than the overlap between larger and smaller males. Both nitrogen and carbon ranges were greater in smaller sharks, and values of CD, MMND and SDNND showed that for the most part, larger sharks had the least trophic diversity, most similar ecologies, and even distribution of trophic niches (Table 1).

**Table 1:**
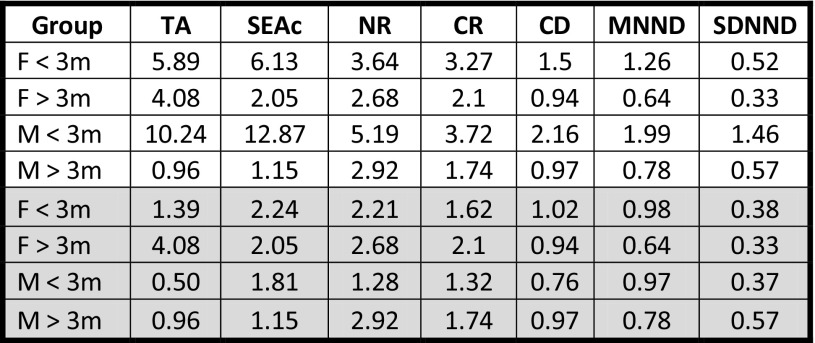
Layman metrics and standard ellipse areas (SEAc) generated for female white sharks less than 3 m in length (F < 3), females over 3 m (F > 3), males less than 3 m (M < 3) and males over 3 m (M > 3)

*TA* convex hull total area, *SEAc* small sample size corrected standard ellipse area, *NR* range of δ^15^N values, *CR* range of δ^13^C values, *CD* mean distance to centroid, *MNND* mean nearest neighbour distance, *SDNND* standard deviation of nearest neighbour distance, *white cells* averaged δ^13^C and δ^15^N data, *grey cells* averaged and outlier-removed δ^13^C and δ^15^N data

**Table 2:**
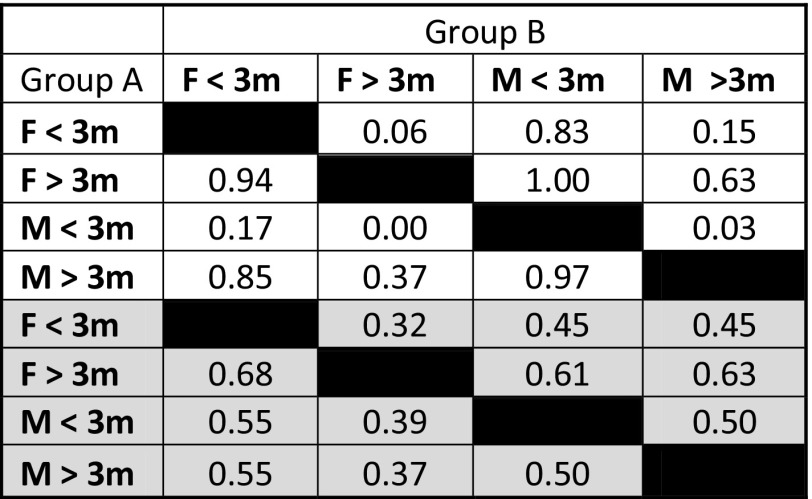
Probability that the standard ellipse area (SEAc) of the isotopic niche of each sex-size demographic group of white sharks (“Group A”) was smaller than the other groups (“Group B”)

Probabilities are for female (F) or male (M) white sharks less than 3 m or over 3 m in total body length

*White cells* averaged δ^13^C and δ^15^N data, *grey cells* averaged and outlier-removed δ^13^C and δ^15^N data

**Table 3:**
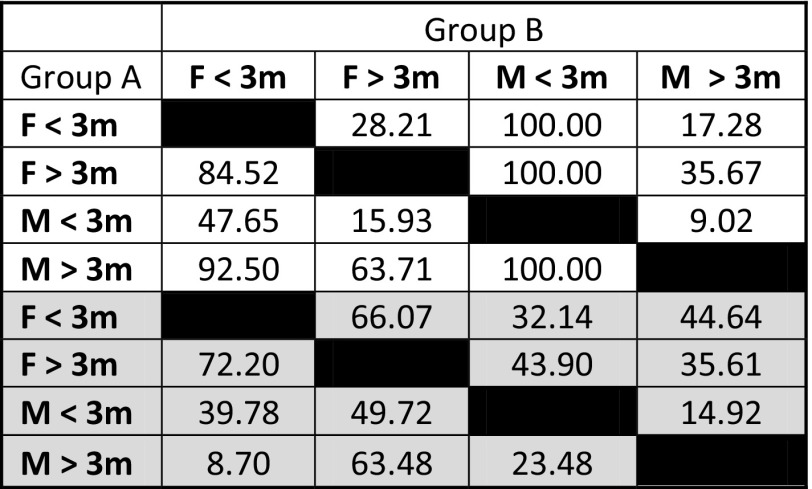
Percentage overlap of SEAc for a sex-size demographic group of white sharks (Group A) with the SEAc of the other groups (Group B)

Percentages are for female (F) or male (M) white sharks less than 3 m or over 3 m in total body length

*White cells* averaged δ^13^C and δ^15^N data, *grey cells* averaged and outlier-removed δ^13^C and δ^15^N data

The isotopic niches of < 3 m sharks were greatly reduced in the outlier-removed dataset (Table 1, Fig. 3c), and Layman metrics became roughly similar across groups (Table 1, Fig. 3d). The biggest change in isotopic niche overlap was between larger males and smaller females, which changed from 92.5 to 8.7% with the removal of outliers. However, females consistently exhibited greater niche overlap than males, and females < 3 m had much greater overlap with males < 3 m than was true for males > 3 m.

## Discussion

Our results reveal isotopic differences between sexes of white sharks. Male sharks exhibited clear change in δ^13^C with increasing shark length, while females retained a more homogenous isotopic niche through ontogeny. Male δ^15^N values also showed a decreasing trend with increasing shark length, and δ^15^N values were significantly related to δ^13^C for outlier-removed shark data. Averaged data revealed differences in niche size between size classes of shark, which were greatly reduced when outliers were removed. Though SIBER sample sizes were comparatively small, we believe that the results can still provide useful insights, especially when interpreted within the context of the available literature.

The change in δ^13^C values with increasing male length, the evident male length differences between clusters based on both δ^13^C and δ^15^N data, and the significant relationship between δ^13^C and δ^15^N overall, are indicative of an ontogenetic change in food web, and potentially a concurrent change in diet, in male sharks. Our δ^13^C results suggest that males either feed further offshore, or in more westerly coastal habitats as they age (Hill et al. 2006; Hill and McQuaid 2008), which could explain the observed relative lack of males caught in KZN, and a paucity of males at the Western Cape in the summer (Cliff et al. 2000; Kock et al. 2013; Towner et al. 2013). Previous studies in South Africa and globally have also shown that white sharks utilise offshore areas more as they age (Boustany et al. 2002; Bonfil et al. 2005; Bruce 2006; Weng et al. 2007; Domeier and Nasby-Lucas 2008; Bonfil et al. 2010; Hussey et al. 2012b; Smale and Cliff 2012; Carlisle et al. 2012; Hoyos-Padilla et al. 2016), but have not detected the male bias evident in our results. While we did not find a significant relationship between male length and δ^15^N, males, and particularly those > 3 m, did show an overall trend for depletion of δ^15^N with increasing length, which may have been weakened by a relatively small sample size. Depletion in δ^15^N has been found previously in the largest white sharks of other studies, and suggests that pelagic prey items are an important part of male diet as they age (Hussey et al. 2012b; Smale and Cliff 2012; Carlisle et al. 2012).

Females did not exhibit the relationships between length and δ^13^C or δ^15^N found in males, which could be due to multiple factors. Satellite tracking and sighting data of South African white sharks indicates that only large individuals cross the Mozambique Basin to Madagascar, with only mature females travelling up to the northern Mascarene Plateau (Cliff et al. 2000; Zuffa et al. 2002; OCEARCH 2016). Our muscle samples represent a relatively slow isotopic turnover rate, and therefore, long-term diet and habitat use (MacNeil et al. 2006), comprising the average isotopic uptake over up to 2 years (Martinez del Rio et al. 2009; Logan and Lutcavage 2010; Hussey et al. 2012a). If females are roaming over a larger area than males, as appears the case in South Africa and as has been found in the North-Eastern Pacific population (Jorgensen et al. 2010; Domeier and Nasby-Lucas 2012), a greater degree of averaging of the δ^13^C signatures of several habitats is likely, resulting in less clear cut trends. Alternatively, the lack of relationships for both δ^13^C and δ^15^N and female shark length could be explained by dietary specialisation, which has been identified in NE Pacific and Australian white sharks (Kim et al. 2012; Pethybridge et al. 2014). Specialisation on piscine prey and/or lack of ontogenetic dietary shift in females is further suggested by the fact that females within the two clusters identified in the outlier-removed data were of the same average length, and that large females consistently exhibited greater isotopic niche overlap with smaller sharks than larger males did. Additionally, females lack a significant ontogenetic change in tooth shape (French et al. 2017) which is reported to facilitate a change in diet from largely fish based, to heavily reliant on marine mammals (Tricas and McCosker 1984; Frazzetta 1988), and greater reliance on fish in the females compared to males studied here is supported by fine-scale habitat use and seasonal abundance of sharks acoustically tagged in False Bay, Gansbaai and Mossel Bay (Kock et al. 2013; Jewell et al. 2013, 2014; Towner et al. 2013, 2016). Lastly, there is evidence of multiple coastal migration strategies in females that may preclude clear isotopic trends. Easterly migrations to the coast of KZN peak either in midwinter or mid-summer, with a capture bias towards females (Cliff et al. 1989; OCEARCH 2016). These peaks coincide respectively with either 1) a mass migration event of *Sardinops sagax* pilchard (the ‘sardine run’; Whitehead et al. 1985) that attracts high densities of the mesopredator prey of white sharks (Cliff et al. 1989; Dudley et al. 2005; Dudley and Cliff 2010), or 2) abundance of high densities of dusky shark (*Carcharhinus obscurus*) and reef manta ray (*Manta alfredi*) prey species (Smale 1991; Dudley et al. 2005; Marshall and Bennett 2010a, b). Females attending the Gansbaai aggregation could be following one of two strategies during summer, either staying at the Western Cape to feed on elasmobranchs and teleosts, or migrating east to take advantage of shark and ray prey availability in Algoa Bay, KZN and Mozambique. Sharks that migrate in midwinter seem likely to be exploiting prey availability associated with the sardine run, be it the sardines themselves (Dudley and Cliff 2010), or the mesopredators that the sardines attract.

While we found overlap between isotopic niches of all demographic groups, similar to other South African white shark diet studies, we also found evidence of expanded and diverse niches in juvenile sharks in comparison to those > 3 m (Cliff et al. 1989; Hussey et al. 2012b; Christiansen et al. 2015), where all our outliers were juveniles. This concords with expanded habitat use found in smaller white sharks in South Africa (Jewell et al. 2013). Christiansen et al. (2015) suggested multiple reasons why South Africa’s young white sharks show such diversity in isotopic signatures, including individual variation, spatial segregation, and maternal influences. In the case of smaller sharks at the Gansbaai aggregation, temporal variation could also play a strong role in their isotopic diversity, representing a function of the time since they undertook the westerly coastal migration for the first time (Cliff et al. 1989, 1996; Ferreira and Ferreira 1996; Dicken 2008; Kock et al. 2013; Towner et al. 2013; Hewitt 2014; Ryklief et al. 2014). Kelp detritus contributes significantly to the coastal food web of South Africa (Bustamante and Branch 1996; Miller and Page 2012), and recorded variation in δ^13^C values of kelp could also partially explain the variation in SIBER niches between juveniles and larger sharks as juveniles make comparatively more use of coastal habitat as opposed to the pelagic or tropical habitats utilised by larger individuals (Cliff et al. 2000; Zuffa et al. 2002; Bonfil et al. 2005; Hussey et al. 2012b; Smale and Cliff 2012; OCEARCH 2016). However, this would not account for the concurrent variation in δ^15^N values found in Christiansen et al. (2015) and this study.

Our results, combined with multifaceted evidence of individual and sexual variation in diet, movement, and foraging strategies in South Africa and globally, suggest that that sex and individual specialisation are key drivers in ecological variation in white sharks, which remain important through ontogeny (Estrada et al. 2006; Hussey et al. 2012b; Carlisle et al. 2012; Kim et al. 2012; Kock et al. 2013; Towner et al. 2013, 2016; Pethybridge et al. 2014; Huveneers et al. 2015; Christiansen et al. 2015). Intraspecific trait variation in a predator population has important implications for community ecology and species conservation (Bolnick et al. 2003, 2011; Schreiber et al. 2011; Mittelbach et al. 2014). In South Africa, the sexes exhibit ontogenetic differences in habitat use, migration patterns and diet, and juvenile sharks have expanded niches compared to larger sharks, which may be the result of multiple factors including specialisation and temporal effects. These sex, age, and individual driven differences should be considered in conjunction with exposure to spatially explicit threats, such as fisheries and pollution, when developing management strategies, and explicitly included in ecological studies of the species.
